# The functional connectivity and neuropsychology underlying mental planning operations: data from the digital clock drawing test

**DOI:** 10.3389/fnagi.2022.868500

**Published:** 2022-09-20

**Authors:** Catherine Dion, Jared J. Tanner, Erin M. Formanski, Anis Davoudi, Katie Rodriguez, Margaret E. Wiggins, Manish Amin, Dana Penney, Randall Davis, Kenneth M. Heilman, Cynthia Garvan, David J. Libon, Catherine C. Price

**Affiliations:** ^1^Department of Clinical and Health Psychology, University of Florida, Gainesville, FL, United States; ^2^Department of Biomedical Engineering, University of Florida, Gainesville, FL, United States; ^3^Department of Physics, University of Florida, Gainesville, FL, United States; ^4^Department of Neurology, Lahey Hospital and Medical Center, Burlington, MA, United States; ^5^Computer Science and Artificial Intelligence Laboratory, MIT, Cambridge, MA, United States; ^6^Department of Neurology, University of Florida, & North Florida/South Georgia Veterans Affairs Medical Center, Gainesville, FL, United States; ^7^Department of Anesthesiology, University of Florida, Gainesville, FL, United States; ^8^Department of Geriatrics and Gerontology, New Jersey Institute for Successful Aging, School of Osteopathic Medicine, Rowan University, Glassboro, NJ, United States; ^9^Department of Psychology, Rowan University, Glassboro, NJ, United States

**Keywords:** neuropsychology, digital technology, digital assessment, executive control, mental planning, basal nucleus of Meynert (BNM), anterior cingulate (ACC)

## Abstract

We examined the construct of mental planning by quantifying digital clock drawing digit placement accuracy in command and copy conditions, and by investigating its underlying neuropsychological correlates and functional connectivity. We hypothesized greater digit misplacement would associate with attention, abstract reasoning, and visuospatial function, as well as functional connectivity from a major source of acetylcholine throughout the brain: the basal nucleus of Meynert (BNM). Participants (*n* = 201) included non-demented older adults who completed all metrics within 24 h of one another. A participant subset met research criteria for mild cognitive impairment (MCI; *n* = 28) and was compared to non-MCI participants on digit misplacement accuracy and expected functional connectivity differences. Digit misplacement and a comparison dissociate variable of total completion time were acquired for command and copy conditions. *a priori* fMRI seeds were the bilateral BNM. Command digit misplacement is negatively associated with semantics, visuospatial, visuoconstructional, and reasoning (*p*’s < 0.01) and negatively associated with connectivity from the BNM to the anterior cingulate cortex (ACC; *p* = 0.001). Individuals with MCI had more misplacement and less BNM-ACC connectivity (*p* = 0.007). Total completion time involved posterior and cerebellar associations only. Findings suggest clock drawing digit placement accuracy may be a unique metric of mental planning and provide insight into neurodegenerative disease.

## Introduction

Mental planning is characterized by the ability to recruit and effectively deploy selected neurocognitive operations necessary to bring a given task to fruition and is critical for the successful completion of many complex activities of daily living such as managing personal finances, organizing work schedules, arranging travel plans, and preparing meals (Owen, 1997; Cohen and Conway, 2007). Disrupted mental planning is often present in older adults with mild cognitive impairment (MCI) and dementia (Cohen et al., 2008). Mental planning involves cholinergic nervous system activity from the basal forebrain (Sarter and Paolone, 2011; Demeter and Sarter, 2013) and frontal and posterior brain regions (Furey et al., 2008a, b; Bracco et al., 2014). Mental planning requires diverse neurocognitive operations including executive abilities, semantic knowledge, and visuospatial operations (Luria, 1966).

The Clock Drawing Test (CDT; Freedman et al., 1994; Libon et al., 1996) is a general cognitive screening measure requiring mental planning for successful completion. The CDT’s command condition requires participants to “*draw the face of a clock, put in all the numbers and set the hands to 10 after 11*,” while the copy condition involves copying a pre-drawn model clock. The CDT command requires the coordination of a number of semantic, visuospatial, and executive functions, while the copy condition primarily revolves around executive functions related to appreciating the visuospatial nature of the model (Price et al., 2011). Comparisons between CDT command and copy conditions with hands set for “*10 after 11*” has been well investigated and show unique patterns for MCI and dementia types (Libon et al., 1996; Price et al., 2011). To date, however, critical aspects of CDT production, such as digit placement accuracy, have strictly relied on visual inspection from trained neuropsychological professionals. The digital version of the CDT (dCDT; Souillard-Mandar et al., 2016) provides a more precise and objective scoring method to quantify digit placement accuracy, broadening the use of the clock drawing as a screening tool for mental planning deficits in a wide variety of clinical settings.

The current project investigated mental planning abilities *via* digital clock drawing digit placement accuracy in command and copy conditions. The first aim was to validate the variable of digit placement accuracy as a measure of mental planning. We examined expected associations between digit misplacement and neuropsychological tests of attention, abstract reasoning, and visuospatial function; cognitive operations thought to underlie planning abilities (Fuster, 2015). As part of this aim, we also compared digit placement accuracy within a sample of individuals with and without MCI. Reduced visual attention is associated with reduced overall cognition (Okonkwo et al., 2008), and adults with MCI display significant mental planning deficits (Yamamoto et al., 2004). We hypothesized MCI participants would display less dCDT placement accuracy, particularly in the command condition given its increased demand on numerous cognitive functions. Second, we explored the neural bases for dCDT digit placement accuracy as a measure of mental planning abilities. Acetylcholine plays a key role in attention and prefrontal functions (Klinkenberg et al., 2011; Fuster, 2015) and is vital for effective mental planning. For this reason, we further hypothesized digit placement accuracy would be associated with functional connectivity from the BNM; a known origin for acetylcholine with vast innervation throughout the brain (Gratwicke et al., 2013). Specifically, we expected that individuals with less digit placement accuracy (or greater digit misplacement) would show less functional connectivity from the BNM. We further expected BNM functional connectivity would be reduced in MCI relative to non-MCI participants due to the general cholinergic reduction in these individuals (Mesulam et al., 1983; Ruberg et al., 1990; Vogels et al., 1990) and that this compromised functional connectivity from the BNM may lead to the cognitive impairment seen in MCI (Whitehouse et al., 1981; Mesulam et al., 1983; Nordberg, 1993; Grothe et al., 2010; Ferreira-Vieira et al., 2016). Lastly, for each examined aim, we compared the cognitive and functional MRI patterns observed with digit placement accuracy to the patterns observed with a common digital clock metric known to associate with numerous areas of cognition: total completion time (Dion et al., 2020). This comparison allowed us to dissociate the cognitive and functional specificity of digit placement accuracy relative to the speed-based metric of total clock drawing time which is known to associate with numerous cognitive domains (Dion et al., 2020).

## Methods

### Participants

Participant data were prospectively acquired through a National Institutes of Health (NIH) funded study approved by the University of Florida Institutional Review Board (IRB). All participants provided written informed consent and the study was conducted in accordance with the Declaration of Helsinki. Inclusion criteria: age ≥55, English as the primary language, and intact instrumental activities of daily living (IADLs; Lawton and Brody, 1969) assessed with both the participant and their caregiver. Exclusion criteria: major neurocognitive, neurodegenerative, or psychiatric disorder at baseline per the Diagnostic and Statistical Manual of Mental Disorders—Fifth Edition (DSM-V; APA, 2013), significant medical illness limiting lifespan, documented learning disorder, seizure disorder, or other neurological illness, <6th-grade education, substance abuse history, major cardiac disease, or chronic medical illness thought to induce encephalopathy.

Participants were screened using the Telephone Interview for Cognitive Status (TICS; Welsh et al., 1993) and during an in-person interview with a neuropsychologist and trained research coordinator to assess comorbidities, anxiety, depression, neuropsychological functioning, and digital clock drawing (Davis et al., 2011). The same examiner administered all test items. fMRI acquisition was completed within 24 h of behavioral testing. Trained raters scored, double scored, and double entered all behavioral data. We defined MCI with Jak and colleagues’ comprehensive criteria using age-adjusted normative data (Jak et al., 2009).

### Neuropsychological measures of interest

Neuropsychological measures were chosen based on their theoretical involvement in mental planning and accurate placement of numbers within the clock face. We converted raw neuropsychological tests score to z-scores using externally published norms (Lezak et al., 2004). Neuropsychological constructs and associated tests:

#### Processing speed

*Digit Symbol, Wechsler Adult Intelligence Scale*—3rd edition (WAIS-III), total correct in 120 s; *Stroop Word Reading*—Word Reading, total correct in 45 s.

#### Working memory

*Letter Number Sequencing, WAIS-III*—total number of correct sequences; *Digit Span (Backward), WAIS-III*—longest span backward.

#### Episodic memory

*Logical Memory, Wechsler Memory Scale—3rd edition (WMS-III)*—total delay recall score; *Hopkins Verbal Learning Test—Revised (HVLT-R)*—delay total recall correct.

#### Naming/lexical access

*Boston Naming Test (BNT)*—total correct out of 60. *Animal fluency*—total correct words in 60 s.

#### Visuospatial operations

*Matrix Reasoning, Wechsler Abbreviated Scale of Intelligence (WASI)*—total correct. *Judgment of Line Orientation (JOLO)*—total number of correct items. *Rey Osterrieth Complex Figure*—copy score.

### Covariates of interest

Age, and cognitive reserve (Stern, 2009). Cognitive reserve, which refers to individual differences in the brain’s ability to offset pathological attacks by relying on premorbid cognitive abilities to maintain cognitive functioning (Stern, 2009), was operationalized by averaging the following estimates of premorbid intelligence: vocabulary knowledge (WASI, vocabulary subtest total correct), word reading using Wide Range Achievement Test (WRAT total score), and years of education (Lezak et al., 2012).

### Clock drawing test

Participants complete a clock drawing to command and copy conditions. The command condition requires participants to “*draw the face of a clock, put in all the numbers, and set the hands to 10 after 11*.” The copy condition requires the participant to draw a clock underneath a pre-drawn model clock.

We captured clock drawing with digital pen technology from Anoto, Inc., which works as a conventional ballpoint pen and measures pen positioning 75 times/s on associated smart article with a spatial resolution of ±0.002 inches. We scored all clock drawings using Clocksketch software, a program developed at MIT, that classifies each pen stroke with over 84% accuracy (Souillard-Mandar et al., 2016). External raters replayed and deconstructed each drawing ensuring accurate scoring (93%–99% inter-rater reliability).

#### Digit misplacement

**Figure 1:**
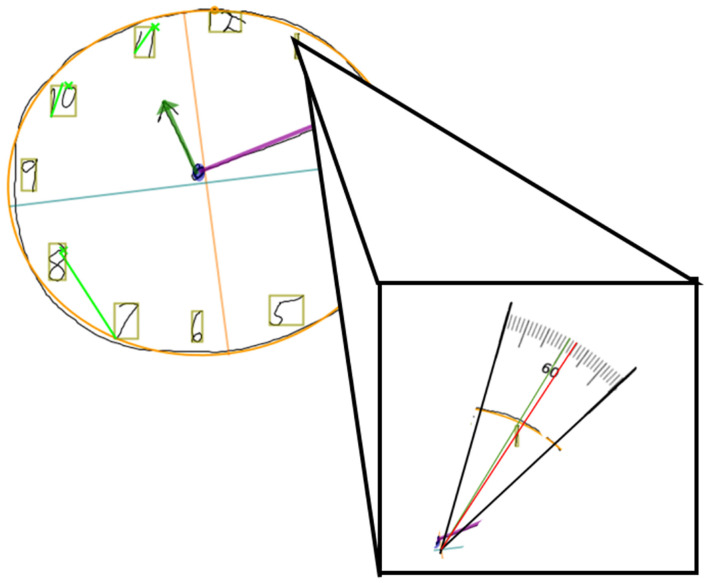
Example of a digital clock drawing, with the clock face of best-fit outlined in orange, the center point indicated by the orange and blue crosshairs, and the digit bounding boxes in tan. Close up of digit 1, which should be centered at 60° (green line) but is slightly misplaced (red line crossing through tan bonding boxes center) at 57.502°. The difference between the red and green values is how misplaced the digit is, indicated as “Digit 1 Ideal Difference”. In this case, Digit 1 Ideal Difference is 60 − 57.502 = 2.498° misplaced.

The Clocksketch software generates a best-fit clock face for the participant’s drawing so that it has a specified “center point” for the clock. The program then spatially identifies each digit and surrounds the strokes of each digit with a bounding box at their widest and highest most points. The center of this bounding box is used to determine a digit’s location in reference to the clock face center point. The software centers a geometric protractor of 360 degrees about the best-fit clock face. An angle of placement for each digit is then calculated from the clock face’s center to the center of each digit’s bounding box. This angle of placement is then subtracted from the “ideal” angle the digit should be placed at, resulting in a degree of misplacement based on the digit’s deviation from the ideal placement (Figure 1). Because of this formulation, we will hereby refer to digit placement accuracy as “digit misplacement” to reference the deviation from the ideal.

We converted all 12 digits’ degrees of misplacement to absolute values to display the distance from the ideal without directionality. A single score to capture overall digit misplacement, termed “Total Misplacement” was then calculated by summing all the absolute ideal differences, as shown by the equation below. This means that *larger* values represent *greater* digit misplacement (a greater value = less-ideally placed digits).


$$Misplacement=\sum_{n=1}^{12}\left(\left|Ideal\;Angel\;of\;Digit\left(n\right)-Actual\;Angel\text{ of }Digit\left(n\right)\right|\right)$$

To ensure precision in measuring mental planning, we excluded data from participants who rotated the article before or during the production of their drawing, omitted a clock face circle, drew more or less than 12 digits and/or repeated digits. The orientation of the article is justification for removal to avoid misplacement reflecting both the degree of misplacement and the degree of improper orientation. Since the clock face circle is required for the program to generate a center point on which to base the ideal placement, digit misplacement could only be calculated for those with a clock face. Perseverations or absence of any of the typical 12 digits may represent confounding impairments with different underlying cognitive constructs than digit misplacement and therefore such data were removed from this sample. The above criteria resulted in the removal of two participants.

#### Total clock drawing time

The Clocksketch software also measures total completion time (TCT). This variable reflects the time taken in seconds to complete all elements of the clock drawing, from the initial pen-article contact until the completion of the last pen stroke. TCT in the command condition positively correlates with traditional neuropsychological measures of processing speed, language, working memory, and declarative memory. In contrast, TCT in the copy condition mainly correlates with processing speed and working memory (Dion et al., 2020). Therefore, we used TCT as a comparison variable with known cognitive correlates to better appreciate the cognitive implications of digit misplacement.

### Statistical analyses—digital clock drawing and neuropsychological tests

Statistical analyses were completed in SPSS v.25 with statistical significance set at *p* < 0.05. Using Spearman correlations, we identified participant demographic covariates. We used separate partial correlations between clock drawing variables (digit misplacement and TCT) and neuropsychological measures controlling for cognitive reserve. We addressed MCI group differences after adjustment for the covariate using analysis of covariance (ANCOVA) and normalized the distribution of clock drawing variables using natural log transformation. We corrected each statistical model for multiple comparisons using a Benjamini-Hochberg correction with FDR set to 0.05. These analyses were then conducted with total completion time for comparison.

### Neuroimaging parameters

Brain MRI data were acquired on a 3T Siemens Verio scanner with an 8-channel head coil. We ran T1-weighted and resting-state fMRI sequences for each participant. T1-weighted data were acquired with the following parameters: TR: 2,500 ms; TE: 3.77 ms; 176 sagittal 1 mm^3^ slices, 1 mm isotropic resolution; 256 × 256 × 176 matrix, 7/8 phase partial Fourier, total acquisition time: 9:22. Resting state fMRI data were acquired with participants’ eyes closed and with the following parameters: TR: 2,000 ms; TE: 30 ms; 36 transverse slices; 3.5 mm^3^ isotropic voxel size, 225 × 225 × 126 matrix. We used Generalized Autocalibrating Partial Parallel Acquisition (GRAPPA) to reduce imaging time (Griswold et al., 2002); total acquisition time: 7:38.

### Resting state fMRI

Preprocessing and quality assurance of functional and structural MRI data were performed using the default pipeline implemented in the CONN Toolbox (19. c^1^; Whitfield-Gabrieli and Nieto-Castanon, 2012), which included functional scan realignment, interleaved slice-timing correction, co-registration to T1w, spatial normalization, and smoothing according to a full-width half-maximum (FWHM) isotropic Gaussian kernel filter of 8 mm. T1w images were then tissue-type segmented into gray matter, white matter, and cerebrospinal fluid (CSF). Nonlinear normalization to Montreal Neurological Institute (MNI152) was then performed. All preprocessing used Statistical Parametric Mapping v.12 (SPM12) software^2^. Functional scans were subjected to artifact and motion outlier identification using the Artifact Detection Toolbox according to conservative settings (95th percentile of the normative sample). These settings identified time points as outliers if movement from a preceding image exceeded 0.5 mm or if the global mean signal intensity exceeded three standard deviations. Seven of 201 participants (3.5%) who had less than 5 min of scan time remaining after time points were identified as outliers and were therefore removed from the analyses. Outlier time points were included as regressors along with principal components delineated from anatomical noise regions (10 components for white matter, five components for CSF) and realignment parameters during a denoising step. Finally, a 0.008–0.09Hz band-pass filter was applied to the functional data (Hallquist et al., 2013).

### fMRI statistical analysis for BNM connectivity

Functional connectivity was calculated using weighted seed-based connectivity (wSBC) maps. The *a priori* seed was the bilateral BNM, a major source of cortical acetylcholine (Johnston et al., 1981; Rye et al., 1984). The BNM region of interest (ROI) was based on a stereotaxic probability map of magnocellular cell groups in the basal forebrain, defined in the standard MNI space (Zaborszky et al., 2008; Chiang-shan et al., 2014). The ROI was then imported into the CONN Toolbox, masking the ROI with gray matter to include only signals coming from inside each participant’s gray matter mask. Next, a mean signal time course for the BNM was calculated. wSBC values were then calculated by correlating the ROI signal time course and the time series of every other voxel in the brain. The correlations in time series between the seed and all other voxels were then Fisher’s r-to-z transformed. Using the CONN Toolbox (Whitfield-Gabrieli and Nieto-Castanon, 2012), we ran general linear models correlating our variables of interest (command and copy digit misplacement and total completion time) with BNM connectivity, while controlling for age and cognitive reserve, which were demeaned before being entered into the general linear model. Clusters of significant connectivity were thresholded using a parametric Gaussian Random Field Theory approach (Worsley et al., 1996), which removes voxels with uncorrected *p* < 0.001 and then keeps only clusters that survived a two-tailed correction for multiple comparisons using an FDR *p* < 0.05. Significant clusters were localized using the Harvard-Oxford Brain Atlas (Desikan et al., 2006) with cerebellar regions from the Automated Anatomical (AAL) Atlas (Tzourio-Mazoyer et al., 2002). Mean r-to-z transformed correlation values for any significant clusters were exported for everyone for additional analyses.

## Results

### Participants

**Figure 2:**
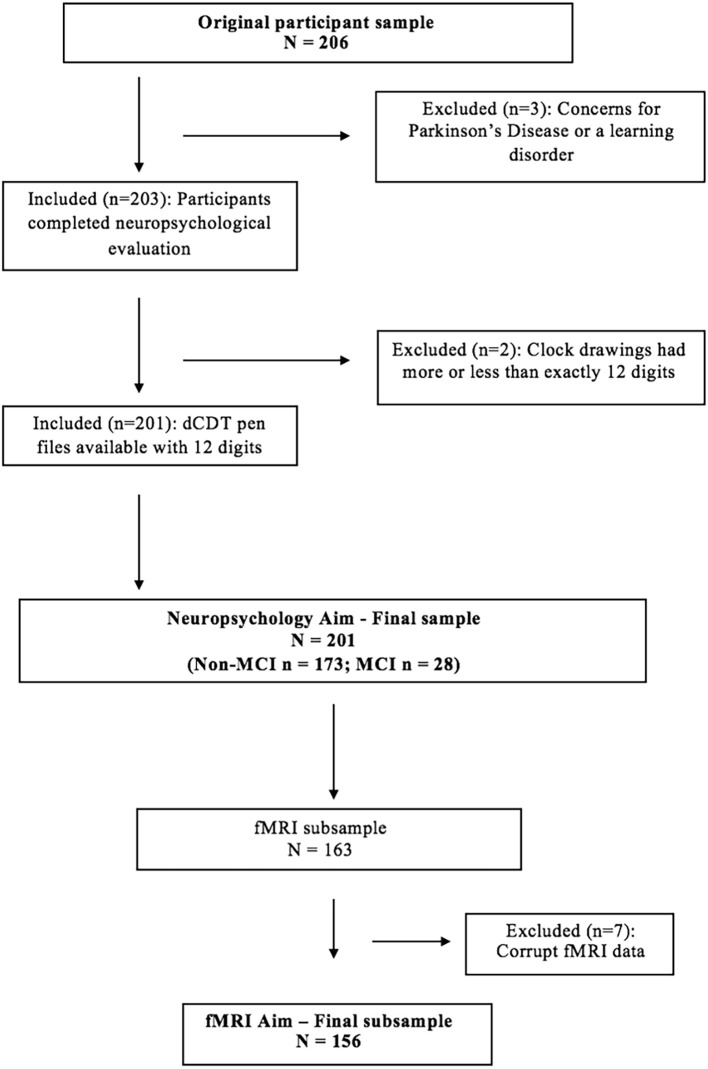
Participant exclusion flow chart.

As part of a prior investigation (Dion et al., 2020), 206 participants completed the neuropsychological protocol and dCDT (Figure 2). We excluded two participants due to concerns revolving around a learning disorder, and one participant due to concerns about Parkinson’s disease (PD). Two additional participants were excluded due to clock drawings having more or less than 12 digits, thus retaining data for 201 participants. Of our retained sample (*n* = 201), 173 participants were classified as non-MCI, while 28 met the criteria for MCI (Jak et al., 2009). Groups did not differ on age or comorbidities; see demographic characteristics by MCI status in Table 1. The MCI group completed fewer years of education resulting in lower cognitive reserve. Of the whole sample (*n* = 201), 163 participants completed neuroimaging, and data from seven participants were removed for poor quality fMRI, for a final sample size with valid neuroimaging data of 156. Of the retained sample with neuroimaging data, 131 were classified as non-MCI, while 25 met the criteria for MCI. Table 2 shows the demographics of the whole sample compared to the neuroimaging subsample.

**Table 1:** Participant characteristics by MCI status.

**Demographic**	**Non-MCI (*n* = 173) Mean ± SD (range)**	**MCI (*n* = 28) Mean ± SD (range)**
Age	68.50 ± 5.80 (55.00–85.00)	70.60 ± 8.12 (60.00–85.00)
Sex (Male : Female)	94 : 77	14 : 14
Race (White : Non-White)	163 : 8	23 : 5
Education	16.33 ± 2.54 (9.00–24.00)	14.27 ± 2.98 (10.00–22.00)*
TICS	38.43 ± 3.41 (29.00–47.00)	34.14 ± 3.59 (26.00–40.00)*
Charlson Comorbidity Index	0.41 ± 0.71 (0.00–4.00)	0.46 ± 0.58 (0.00–2.00)
Cognitive Reserve	0.58 ± 0.58 (-1.25–1.90)	0.03 ± 0.64 (-0.90–1.58)*
STAI-State	27.88 ± 8.02 (20.00–59.00)	30.29 ± 7.37 (20.00–44.00)
STAI-Trait	29.82 ± 7.87 (20.00–55.00)	32.92 ± 7.51 (21.00–48.00)
BDI-II	4.39 ± 5.22 (0.00–30.00)	5.75 ± 4.62 (0.00–17.00)

Note. TICS, Telephone Interview for Cognitive Status; STAI, State-Trait Anxiety Inventory; BDI-II, BeckDepression Inventory—2nd Edition. *Indicates a statistically significant difference (*p* > 0.05).

**Table 2:** Participant characteristics.

**Demographic**	**Total (*n* = 201) Mean ± SD (range)**	**Imaging Subsample (*n* = 156) Mean ± SD (range)**
Age	68.79 ± 6.19 (55.00–85.00)	68.96 ± 6.48 (55.00–85.00)
Sex (Male : Female)	110 : 91	74 : 82
Race (White : Non-White)	188 : 13	143 : 13
Education	16.04 ± 2.69 (9.00–24.00)	15.97 ± 2.78 (9.00–24.00)
TICS	37.82 ± 3.74 (26.00–47.00)	37.55 ± 3.84 (26.00–47.00)
Charlson Comorbidity Index	0.42 ± 0.69 (0.00–4.00)	0.42 ± 0.70 (0.00–4.00)
Cognitive Reserve	0.50 ± 0.62 (-1.25–1.90)	0.51 ± 0.62 (-1.25–1.90)
STAI-State	28.18 ± 7.96 (20.00–59.00)	28.44 ± 8.42 (20.00–59.00)
STAI-Trait	30.21 ± 7.88 (20.00–55.00)	30.39 ± 8.17 (20.00–55.00)
BDI-II	4.56 ± 5.16 (0.00–30.00)	5.28 ± 5.50 (0.00–30.00)

Note. TICS, Telephone Interview for Cognitive Status; STAI, State-Trait Anxiety Inventory; BDI-II, Beck Depression Inventory—2nd Edition.

### Digit misplacement and neuropsychological test performance (whole sample, *n* = 201)

Command: *Greater* digit misplacement was associated with worse performance on the BNT (*r* = −0.319, *p* < 0.001), Matrix Reasoning (*r* = −0.318, *p* < 0.001), Rey Complex Figure Copy (*r* = −0.207, *p* = 0.015), and JOLO (*r* = −0.255, *p* = 0.002).

Copy: *Greater* digit misplacement associated with *poorer* performance on the Matrix Reasoning (*r* = −0.269, *p* < 0.001), JOLO (*r* = −0.198, *p* = 0.019), and Rey Complex Figure Copy (*r* = -0.205, *p* = 0.015; Table 3).

**Table 3:** Partial correlations between digit clock drawing variables (Command and Copy) and neuropsychological tests of interest.

		**Total Digit Misplacement**		**Total Completion Time**	
		**Command *r* *p*-value**	**Copy *r* *p*-value**	**Command *r* *p*-value**	**Copy *r* *p*-value**
Processing Speed	Digit Symbol (WAIS-III)	−0.108 0.207	−0.122 0.153	−0.398 <0.001*	−0.444 <0.001*
	Stroop Word Reading	−0.055 0.520	−0.011 0.895	−0.237 0.005*	−0.245 0.004*
Working Memory	Letter Number Sequencing (WAIS-III)	−0.174 0.040	0.052 0.542	−0.322 <0.001*	−0.200 0.018*
	Digit Span, backward (WAIS-III)	−0.127 0.135	−0.039 0.645	−0.184 0.030*	−0.073 0.393
Episodic Memory	Logical memory (WMS-III)	−0.105 0.219	−0.117 0.171	−0.312 <0.001*	−0.211 0.013*
	Hopkins Verbal Learning Test—Revised	−0.161 0.059	−0.134 0.115	−0.157 0.065	−0.150 0.079
Language	Boston Naming Test	−0.319 <0.001*	−0.077 0.368	−0.158 0.063	−0.011 0.899
	Animal Fluency (COWA)	−0.033 0.696	−0.175 0.039	−0.172 0.043	−0.067 0.431
Visuospatial Operations	Matrix Reasoning (WASI)	−0.318 <0.001*	−0.269 <0.001*	−0.113 0.185	−0.086 0.314
	Judgement of Line Orientation	−0.255 0.002*	−0.198 0.019*	−0.074 0.387	−0.025 0.773
	Rey Osterrieth Complex figure—Copy	−0.207 0.015*	−0.205 0.015*	0.002 0.986	0.028 0.742

Note. *Denotes partial correlation meeting Benjamini-Hochberg’s critical threshold for significance.

### Total completion time and neuropsychological test performance (whole sample, *n* = 201)

Command: *Longer* or slower completion time negatively and significantly correlated with lower scores on Digit Symbol (*r* = −0.398, *p* < 0.001), Stroop Word Reading (*r* = −0.237, *p* = 0.005), Letter Number Sequencing (*r* = −0.322, *p* < 0.001), Logical Memory (*r* = −0.312, *p* < 0.001), and Digit Span Backward (*r* = −0.184, *p* = 0.030).

Copy: *Longer* or slower completion time negatively and significantly correlated with lower scores on Digit Symbol (*r* = −0.444, *p* < 0.001), Stroop Word Reading (*r* = −0.245, *p* = 0.004), Letter Number Sequencing (*r* = −0.200, *p* = 0.018), and Logical Memory (*r* = −0.211, *p* = 0.013; Table 3).

### Digit misplacement and MCI group differences (non-MCI *n* = 173, MCI *n* = 28)

Command: Controlling for cognitive reserve, there was a significant group difference *F*_(1,186)_ = 21.08, *p* < 0.001, *η*^2^ = 0.103, such that the MCI group displayed greater digit misplacement than non-MCI peers. On average, individuals with MCI exhibited 43 degrees of greater misplacement than non-MCI peers (MCI Mean = 112.18, SD = 63.08; non-MCI Mean = 68.76, SD = 32.59).

Copy: Controlling for cognitive reserve, there was a significant group difference *F*_(1,187)_ = 6.99, *p* = 0.037, *η*^2^ = 0.023, such that the MCI group displayed greater digit misplacement than non-MCI peers. On average, individuals with MCI exhibited 19 degrees of greater digit misplacement than non-MCI peers (MCI Mean = 84.34, SD = 37.55; non-MCI Mean = 65.26, SD = 25.76).

### Resting state fMRI: seed-based connectivity from bilateral BNM to whole brain (neuroimaging subsample *n* = 156)

**Figure 3:**
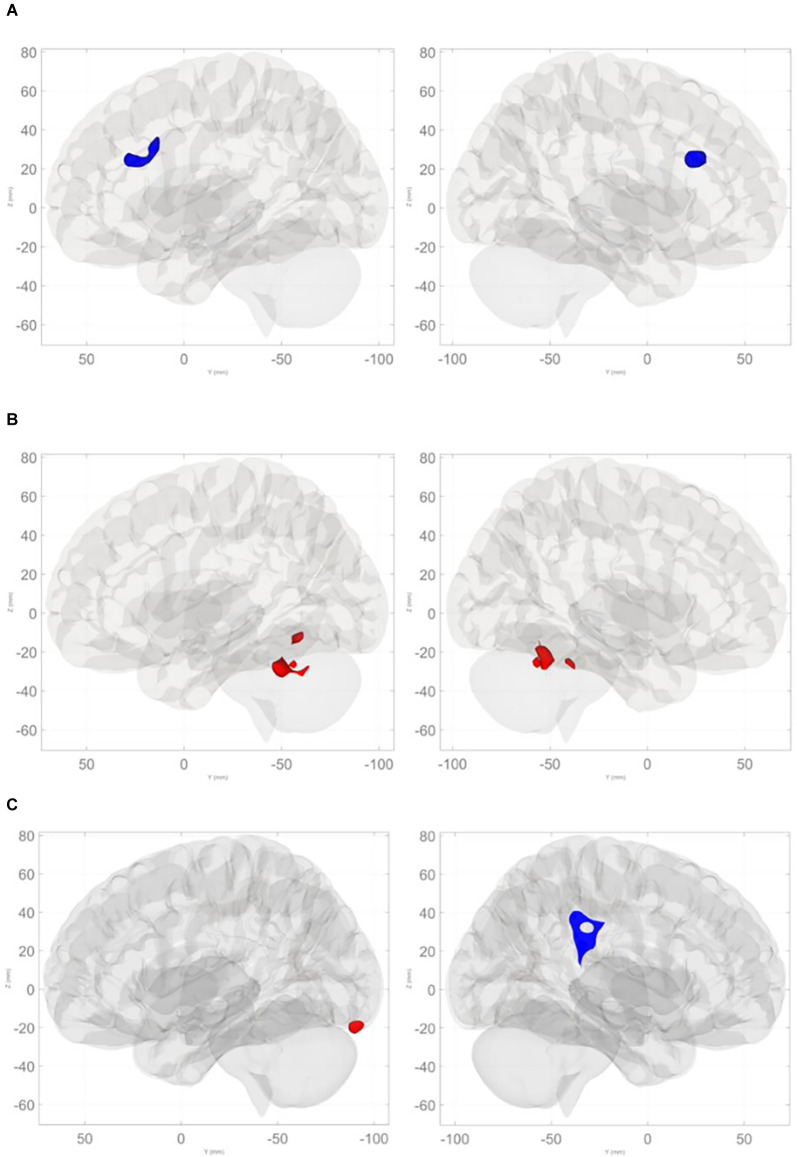
**(A)** Negative relationships between command total digit misplacement and basal nucleus of Meynert connectivity with the anterior cingulate cortex. **(B)** Positive relationships between command total digit misplacement and basal nucleus of Meynert connectivity with cerebellum (anterior lobe, superior posterior lobe, and vermis). **(C)** Negative relationships between command total completion time and basal nucleus of Meynert connectivity with right supramarginal gyrus and contiguous regions, and positive associations with left occipital pole and the left superior posterior lobe of the cerebellum.

Command Digit Misplacement: After controlling for age and cognitive reserve, significant negative associations were found between BNM connectivity with the anterior cingulate cortex (ACC; FDR-corrected *p* = 0.0002; Table 4 and Figure 3A) such that greater digit misplacement was observed in association with less BNM-ACC connectivity. Copy Digit Misplacement: Statistically not significant.

**Table 4:** Clusters demonstrating significant associations between BNM connectivity and digital clock variables.

**Clock variable**	**Cluster**	**Location (x, y, z)**	**Regions**	**# of voxels**	***t*-value**	**Size FWE *p***	**Size FDR *p***
Command	1	+02, +24, +24	anterior cingulate cortex	284	−5.04	0.0002	0.0002
Digit			L paracingulate gyrus	18			
Misplacement			R paracingulate gyrus	13			
Command	1	−08, −60, −14	R cerebellum 4/5	78	5.74	0.0001	0.0003
Total			R cerebellum 6	74			
Completion			L cerebellum 4/5	36			
Time			vermis 4/5	34			
			L cerebellum 6	23			
			vermis 8	18			
			vermis 6	12			
			Right 3	7			
			vermis 9	7			
	2	−22, −48, −28	L cerebellum 6	119	5.36	0.0015	0.0018
			L cerebellum 4/5	40			
			L cerebellum crus 1	2			
Copy	1	+66, −30, +24	R posterior supramarginal gyrus	123	−5.00	0.0004	0.0003
Total			R anterior supramarginal gyrus	91			
Completion			R parietal operculum	19			
Time			R posterior superior temporal gyrus	6			
			R planum temporale	2			
	2	−06, −90, −18	L occipital pole	20	4.98	0.0392	0.0128
			L lingual gyrus	16			
			L cerebellum crus 1	14			
			L cerebellum crus 2	11			
			L fusiform gyrus	4			
	3	−56, −34, +30	L anterior Supramarginal gyrus	69	−4.38	0.0492	0.0128
			L parietal operculum	21			
			L posterior supramarginal gyrus	20			

Note: Regions are labeled according to the Harvard-Oxford Brain Atlas with cerebellar regions coming from the Automated Anatomical (AAL) Atlas.

Command Clock Drawing Total Completion Time: there were significant associations involving BNM connectivity such that longer command total completion time was associated with greater BNM connectivity with bilateral anterior and superior posterior lobules and the vermis of the cerebellum (FDR-corrected *p* < 0.002; see Table 4 and Figure 3B). Copy Clock Drawing Total Completion Time: there were significant associations involving BNM connectivity such that a longer total completion time was associated with less BNM connectivity with right supramarginal gyrus and contiguous regions (FDR-corrected *p* = 0.0003). Longer time to completion is associated with greater connectivity with the left temporal/occipital region and the left superior posterior lobule of the cerebellum (FDR-corrected *p* = 0.0128; see Table 4 and Figure 3C).

### MCI vs. non-MCI resting state connectivity (non-MCI *n* = 131, MCI *n* = 25)

**Figure 4:**
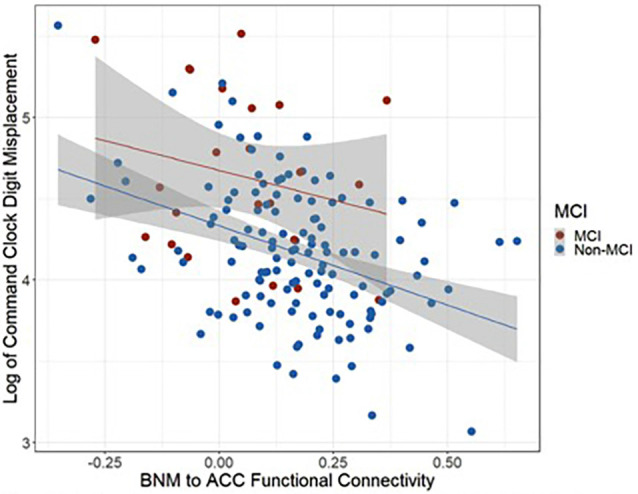
Scatter plot representing the relationships between BNM to ACC functional connectivity and command digit misplacement.

Controlling for age and cognitive reserve, MCI and non-MCI groups significantly differed on BNM-ACC connectivity such that the MCI group (Mean = 0.05, SD = 0.16) showed lower BNM-ACC connectivity than the non-MCI group (Mean = 0.17, SD = 0.17; *t*_(150)_ = 2.35, *p* = 0.0202). Both groups displayed similar relationships between BNM-ACC connectivity and command digit misplacement (see Figure 4). Wilcoxon rank sum tests showed no group differences in command total time to completion or copy total time to completion with BNM connectivity values (BNM to the five clusters, see Table 4). *P* values > 0.456.

## Discussion

Findings suggest clock drawing digit misplacement has expected associations with cognitive metrics and functional connections to the Basal Nucleus of Meynert (BNM). Command digit misplacement is associated with neuropsychological metrics of semantic knowledge, abstract reasoning, visuospatial operations, and visuoconstructional operations with analogous results in the copy condition. These findings extend upon prior research reporting upon neuropsychological constructs of the CDT (Libon et al., 1996; Cosentino et al., 2004; Price et al., 2011; Dion et al., 2020). Further, digit misplacement showed a unique association with BNM-ACC resting state connectivity such that greater digit misplacement was associated with less BNM-ACC connectivity. Research suggests the ACC plays an evaluative role in monitoring behavioral errors and adapting accordingly (Valenstein et al., 1987; Botvinick et al., 2001, 2004). Reduced digit accuracy may therefore represent reduced connectivity from the BNM to the ACC.

The current study also identified MCI and non-MCI group differences such that older adults with MCI showed greater digit misplacement and lower functional connectivity from BNM. The MCI findings extend upon research demonstrating how neurodegenerative conditions such as Alzheimer’s disease (AD) are associated with forebrain function and altered afferent cholinergic projection into cortical and subcortical regions (Kuhl et al., 1996). The MCI and non-MCI group differences are also consistent with prior research showing that, in comparison to healthy controls, older adults with MCI display lower functional connectivity from BNM to the insula/claustrum, involved in the integration of various cortical inputs (Li et al., 2017). ACC input to the claustrum is thought to play a criterial role in modulating visuospatial operations and impulsivity in animal models (Robbins, 2002; Li et al., 2017; White and Mathur, 2018). Further, Cantero and colleagues demonstrated that in amnestic MCI, BNM atrophy is associated with structural changes in their innervated regions (Cantero et al., 2017).

By contrast, the functional networks associated with total completion time suggest a different cognitive-neuronal fMRI profile. First, faster copy clock drawing was associated with increased connectivity from BNM to left and right lateral supramarginal gyri. Research suggests the supramarginal gyrus is involved in a wide variety of tasks including requiring cross-modal cognitive operations (Butters et al., 1970; Geschwind, 1974) as well as tasks assessing visuomotor abilities and mental flexibility of motor action (Hanakawa et al., 2008). Second, for the command, and to a lesser extent the copy condition, slower clock drawing is associated with *increased* BNM-cerebellar connectivity, particularly in regions IV through VI, including the vermis. Since cerebellar acetylcholine comes primarily from brainstem nuclei afferents (Zhang et al., 2016), increased connectivity with the BNM may indicate abnormal organization, since the cerebellum is involved in motor, cognition, emotion, and vestibular functions (Schmahmann and Sherman, 1998; Manto and Mariën, 2015). Prior work has linked gray matter volume in the posterior lobules to processing speed in multiple sclerosis (Moroso et al., 2017). Task-based functional neuroimaging indicates increased anterior cerebellar activity in MCI (for a summary see Jacobs et al., 2018) and associations with lobular volumes suggests associations with executive function but also visuomotor coordination and memory (Kansal et al., 2017). Lastly, we found no group differences between MCI and non-MCI groups in command total time to completion or copy total time to completion with BNM connectivity values (BNM to the five clusters). These findings suggest these relationships appear to be somewhat independent of MCI status, unlike digit misplacement and BNM-ACC connectivity. We encourage additional research examining subtle behavior features of clock drawing and MCI profiles.

We recognize study limitations. First, the MCI sample was small, limiting statistical power. Thus, the MCI analyses may have been underpowered to detect other meaningful differences across groups. Given this small sample, we chose not to investigate potential differences across MCI phenotypes (i.e., single/multiple-domain amnestic MCI, dysexecutive MCI, mixed MCI). Second, our MCI group completed fewer years of education, likely influencing our measure of cognitive reserve, hence our statistical correction for cognitive reserve. Third, the study has limitations including an obvious lack of ethnic and educational diversity. Given this underrepresentation, we did not investigate differences across ethno-racial or education groups. Future studies need to expand this research to ethno-racially, geospatially, and more educationally diverse samples. Fourth, since the dCDT scoring program fits the best-fit ellipse to the clock face as the first step in deriving digit misplacement, highly asymmetrical clocks may require additional statistical corrections. Additionally, to create a single continuous measure of digit misplacement for the entire drawing, misplacement for all 12 digits was summed. For this reason, we included clock data only from those who drew 12 digits. Our results should, therefore, be interpreted in such context and may not be generalizable to highly impaired clock drawings containing more or less than 12 digits. Future research should include clock drawings of individuals with different forms of cognitive impairment, such as vascular dementia, AD, and surgical samples. Another potential limitation is the size of the BNM region of interest. Although the BNM region of interest (ROI) was based on a stereotaxic probability map of magnocellular cell groups in the basal forebrain, defined in the standard MNI space (Zaborszky et al., 2008; Chiang-shan et al., 2014), we note that the ROI is larger than the BNM proper. Consequently, data acquired are not fully specific to the BNM and include some of the surrounding gray matter. Lastly, we used the variable of total time to completion only for comparison purposes with digit misplacement. Previous work from our team shows that individuals with MCI take significantly longer to draw their clocks to command (i.e., on average 10 s longer) relative to non-MCI peers (Dion et al., 2020). Future studies should investigate the neurobiological underpinnings of total time to completion.

Despite limitations, our investigation has a number of strengths. The study examined two distinct subtle behavioral features captured *via* digital technology methods. Digital technology cognitive assessment and rapid behavioral capture will be increasingly due to the need for community-based cognitive screening (Diaz-Orueta et al., 2020). The scientific questions were based on theoretical constructs of mental planning and functional connectivity involving the BNM. The study examined an objective metric of digit misplacement (which is in contrast to the longstanding subjective measurement of digit placement accuracy based on visual scoring). This novel approach reduces the potential for scoring error and removes scoring subjectivity, thereby increasing accessibility for clinical use. Further, the metric was compared to a more common dCDT metric of total time to completion, thereby providing us with insight into the unique functional connectivity associations of the digit misplacement metric. Our large sample of non-demented older adults is also well-characterized based on a comprehensive neuropsychological protocol, allowing for high confidence in the correlations between neuropsychological domains and neuroimaging. We additionally classified our MCI sample based on rigorous published criteria validated through neuroimaging and pathology reports (Jak et al., 2009), thus allowing for meaningful results/interpretation in a smaller sample.

Study findings add to the existing literature documenting the advantages of appreciating subtle behavioral nuances and their contribution to our understanding of brain-behavior relationships. The incorporation of digital technology of clock drawing allows for the identification of subtle behaviors, such as digit misplacement, that could otherwise not accurately be categorized *via* traditional article and pencil testing (Emrani et al., 2020, 2021; Davoudi et al., 2021; Dion et al., 2021). Digit misplacement appears to assess a unique aspect of cognition in older adults and may help identify those with compromised attentional networks specific to the BNM and ACC. The comparison variable of total time to completion associates with multiple cognitive domains including processing speed (Dion et al., 2020) and, in the current study, involved posterior and cerebellar associations. Within rapid screening settings for community interventions (e.g., Amini et al., 2019), these rapid clinical metrics may have a unique advantage. We encourage the investigation of the predictive value of digitally acquired metrics from tests such as clock drawing on brain metrics and/or cognitive status. Data will guide future investigations addressing digitally acquired clock drawing profiles in neurodegenerative disorders.

## Data Availability Statement

The raw data supporting the conclusions of this article will be made available by the authors, without undue reservation.

## Ethics Statement

The studies involving human participants were reviewed and approved by University of Florida Institutional Review Board. The patients/participants provided their written informed consent to participate in this study.

## Author Contributions

Conceptualization and methodology: CD, JT, EF, DL, and CP. Data acquisition: KR and JT. Data curation: CD, JT, EF, AD, and MA. Writing—original draft preparation: CD and EF. Writing—review and editing: CD, JT, EF, MW, DP, RD, DL, KH, CG, and CP. Supervision: DL and CP. All authors contributed to the article and approved the submitted version.

## Funding

This work was supported by the National Institute of Health (grant nos. R01AG055337; P50AG047266; R01NR014181; R01NS082386; UL1R001427; K07AG066813); and the National Science Foundation (grant no. 1404333).

## Footnotes

^1^ http://web.conn-toolbox.org/home

^2^ http://www.fil.ion.ucl.ac.uk/spm/software/spm12/
